# Immediate Impacts of Wildfires on Ground-dwelling macroinvertebrate Communities under Stones in Mediterranean Oak Forests

**DOI:** 10.1007/s00267-024-02006-z

**Published:** 2024-06-21

**Authors:** João R. L. Puga, Francisco Moreira, Jan J. Keizer, Nelson J. C. Abrantes

**Affiliations:** 1 CESAM—Centre for Environmental and Marine Studies, Department of Environment and Planning, University of Aveiro, Aveiro, Portugal; 2 Research Center in Biodiversity and Genetic Resources (CIBIO-InBIO), University of Porto, Porto, Portugal; 3 GEOBIOTEC—Geobiosciências, Geoengenharia e Geotecnologias, Department of Geosciences, University of Aveiro, Aveiro, Portugal; 4 CESAM—Centre for Environmental and Marine Studies, Department of Biology, University of Aveiro, Aveiro, Portugal

**Keywords:** Wildfires, Invertebrates, Post-fire effects, Oak, Stones, Mediterranean

## Abstract

Wildfires are considered a major disturbance to forest ecosystems in the Mediterranean countries of Southern Europe. Although ground-dwelling macroinvertebrates are crucial to many soil functions, there is a fundamental lack of understanding of how wildfires impact this community in the immediate term and of the role of stones in their survival. Hence, in the present study we assessed the immediate effects of wildfires in the ground-dwelling macroinvertebrate community found under stones by comparing communities in burnt and non-burnt Mediterranean oak forests. Our results revealed that stones allowed the survival of many taxa in the burnt area. However, abundance, richness, diversity, and equitability per stone were significantly lower at the burnt than unburnt sites. Furthermore, the results also showed that richness and abundance increased significantly with increasing stone depth and area, both at the burnt and unburnt sites. Significant changes at the trophic level were observed in the burnt area comparing to the unburnt, particularly a decline in predators. No significant differences were identified concerning habitat associations among taxa. Overall, this study stressed the role of stones as microhabitats and refuge for the ground-dwelling macroinvertebrate community during wildfires.

## Introduction

Native oak forests play a crucial role in fostering biodiversity, serving as valuable habitats for numerous plants and animals (Silva 2007; Calviño-Cancela et al. 2012). However, human activities such as agriculture, wood harvesting, grazing, and urban expansion have led to their decline over the centuries. In Europe, very few native forests remain (Depauw et al. 2019), and Portugal is no exception. Oak forests, once the dominant native forest in Portugal for millennia, have significantly diminished due to human-induced changes throughout the centuries (Silva 2007). This is especially true for deciduous oak tree forests (*Quercus faginea* and *Quercus pyrenaica*), which now exist in small patches across the country, representing only 3% of the national forest area (IFN6 2015). These native forests have progressively transformed into agricultural areas, now abandoned, and more recently replaced with plantation forests, such as maritime pine and eucalyptus (Silva 2007). These transformations, coupled with inadequate land and forest management, have been identified as one of the major reasons for the increasing wildfire susceptibility in the Mediterranean basin (Colantoni et al. 2020).

While wildfires are a natural phenomenon that has shaped ecosystems for millennia, the wildfire regime has intensified, presenting a global challenge (Pausas et al. 2008; Moreira et al. 2011). In recent decades, Portugal has become one of the countries most affected by wildfires, with an annual average burnt area of 140,000 ha (Doerr and Santín 2013; Rego and Silva 2014; San-Miguel-Ayanz et al. 2017). Future climate predictions indicate a worsening situation in this region of the world (Flannigan et al. 2013).

Given that natural forests globally support more than half of the known terrestrial animal species (Brockerhoff et al. 2008; Yekwayo et al. 2016), understanding the effects of fires on these ecosystems is necessary to invert the loss of biodiversity. Invertebrates are considered good indicators of soil disturbance due to their ecological relevance in processes such as nutrient cycling, organic matter decomposition, mineralization, and their role in food web dynamics (Bedano et al. 2016; Pedley et al. 2016; Swart et al. 2017; Abrantes 2018). However, the impacts of fire on most invertebrate groups are poorly understood (Swengel 2001; Doblas-Miranda et al. 2009; Saunders et al. 2021). Many studies on invertebrate fire ecology focus on broader taxonomic or functional groups (Moretti et al. 2006; Caut et al. 2013) or specific taxa (York 1999; Kiss et al. 2004; Teasdale et al. 2013). These approaches can oversimplify or broaden the collected information, often neglecting ecological interactions, such as specific microhabitats’ community responses to post-fire conditions in a given area. Additionally, many studies often use single sampling methods, relying on capturing broader community spectrums, leading to biased interpretations of results (Swengel 2001; Abensperg-Traun and Steven 1995).

Direct mortality, community shifts, species displacement, food scarcity after a fire, and habitat loss are among the most common adverse effects of wildfires on faunal communities (Sgardelis et al. 1995; Moreira et al. 2010; Kim and Holt 2012, New 2014), often resulting in decreased biodiversity. However, contradictory findings have been reported in this regard (Zaitsev et al. 2016). Some studies suggest that ground-dwelling invertebrates are negatively affected by fire immediately afterward up to several years later (Trucchi et al. 2009; García-Domínguez et al. 2010; Elia et al. 2012; Verble-Pearson and Yanoviak 2014). In contrast, other studies report that ground-dwelling invertebrates (ranging from order to species level) are not affected by fire or recover rapidly (Siemann et al. 1997) and may even benefit from fire (Moretti et al. 2006; Jacobs et al. 2015).

Some groups of invertebrates start recolonizing burnt areas days after the fire, depending on their resilience to fire and behaviour, as well as characteristics of the fire itself (Gongalsky et al. 2012; Kim and Holt 2012). It is widely accepted that post-fire recolonization of the invertebrate community starts from nearby unburnt areas and unburnt pockets of forest within the burnt areas or pockets with low fire severity (Zaitsev et al. 2014). However, habitats such as burnt trees can often safeguard some species from the direct impacts of fire, for example saproxylic beetles (Ulyshen et al. 2010). Hence, burnt trees and other natural structures such as stones or crevices, may be of great relevance to post-fire recolonization processes. Nevertheless, information related to the ecological role of natural structures and micro-habitats is scarce, particularly during natural phenomenon such as wildfires (Abbott and Maitre 2010; Ross et al. 2017).

In this study we investigated the immediate effects of wildfires on the composition of ground-dwelling community in Mediterranean oak forests, focusing on the role of stones as safeguard microhabitats. Ground stones are natural structures abundant in many ecosystems, resistant to most destructive natural phenomena, and used by several groups of animals, making them a potentially important shielding microhabitat against those phenomena. The proposed hypotheses are: (i) ground stones safeguard their associated community from direct fire effects; (ii) the feeding behaviour and habitat preference of the ground-dwelling macroinvertebrate community change with the wildfire occurrence; (iii) the area and depth of ground stones play an important role in the survival and composition of the ground-dwelling macroinvertebrate community.

## Materials & Methods

### Study Area

**Fig. 1:**
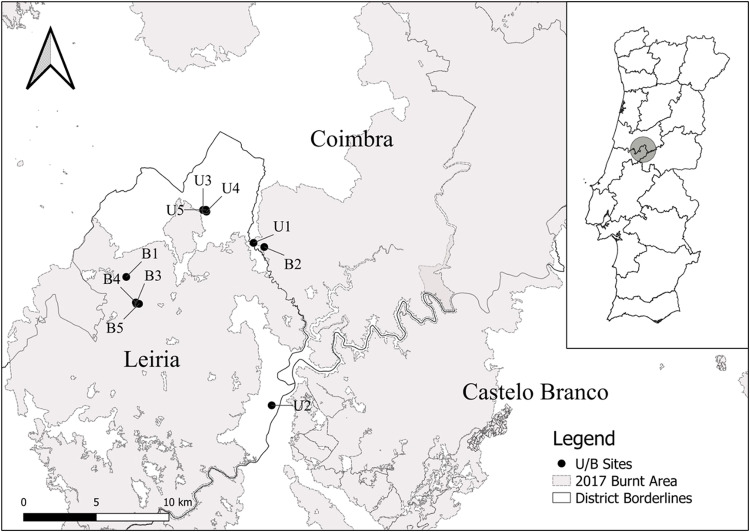
Location of the study area and sampling sites. Unburnt sites (U): U1, U2, U3, U4, U5; Burnt sites (B): B1, B2, B3, B4, B5

The study took place in the municipalities of Pedrogão Grande (129 km^2^) and Castanheira de Pêra (67 km^2^) in the Leiria District, located in Central Portugal, in an area affected by one of the most dramatic and devastating wildfires in the country that occurred in June 2017. This wildfire affected a total area of 45000 ha, located mostly within Pedrogão Grande and Castanheira de Pêra municipalities, being classified as a medium-to-high severity fire (ICNF 2017) (Fig. 1). The climate of area is identified as temperate with hot and dry summer (Csa) and temperate with dry or temperate summer (Csb) according to the Köeppen-Geiger classification system, with an average annual temperature of 14.9 °C and an average annual rainfall of 1010 mm. The soils are mainly derived from schist but locally from granite and quartz. The characteristic climax vegetation is a forest dominated by cork oak (*Quercus suber*), black oak (*Quercus pyrenaica*), and strawberry tree (*Arbutus unedo*) (Costa et al. 1998), but it is nowadays reduced to small patches. At present, mono-specific plantations of eucalypt (*Eucalyptus globulus*) and maritime pine (*Pinus pinaster*) are now the main forest types in the region (Oliveira et al. 2017).

### Sampling Design

The available CORINE land cover and Google Satellite maps (Google Earth Pro 2017) were combined with the burnt area map (EFFIS 2017), to pre-select stands of burnt and unburnt oak forest in the region (Fig. 1). Field inspection of these sites then resulted in the selection of a total of five burnt (B) and five unburnt (U) study sites. Sites were at least 1 km apart and were similar in terms of elevation (500–800 m a.s.l.), orientation (S-SW), parent material (schist), tree age (>50 years), land use, and, in the case of the burnt sites, fire severity using soil and vegetation assessments (Parson et al. 2010). The number of sites was limited by low availability of suitable areas due to their growing scarcity and by the time since fire for sample collection in the burnt area. Experimental feasibility and statistical considerations were used to select the site to provide a robust data analysis. The sampling of the ground-dwelling macroinvertebrate community took place less than one week after the wildfire, and the selected sampling sites corresponded to mixed cork and black oak forest patches on former agricultural lands abandoned several decades ago.

Each site was distanced at least 1 km apart and integrated 3 randomly selected linear transects with 50 m, each divided into 11 points separated by 5 m. At each sampling point, the nearest non-rolling stone that was larger than roughly 50 cm^2^ but could still be lifted by hand was selected as sampling unit, so that a total of 330 stones was sampled across all 10 sites. Under the assumptions that each stone had been unmoved for a long period of time and because of that the community under it was already established, each stone was considered and independent sample.

### Stone Characterization and Ground Cover

The maximum length, width (to the nearest 1 cm), and depth into the soil were measured for each sampled stone. Furthermore, around each stone, a plot of 1 m by 1 m was laid out to visually estimate the percentage of ground cover of stones (U = 23%; B = 31%), bare soil (U = 0%; B = 55%), litter (U = 58%; B = 0%), and vegetation (U = 19%; B = 14%). A fire severity assessment was also applied to ground cover and ash colour and depth for each 1 m by 1 m plot around each stone in the burnt area, according to Parson et al. (2010).

### Ground-dwelling Macroinvertebrate Sampling and Identification

Immediately after removal a selected stone, all living and dead ground-dwelling macroinvertebrates were collected using aspiration (pooter) and direct collection by hand. All collected organisms were then immediately preserved in ethanol.

In the case of ant and termite nests with living specimen, a specific sampling approach was used: (a) for smaller taxa the sampling consisted of 1-min aspiration; (b) for bigger taxa only a few specimens were captured by hand. All collected organisms were also preserved in ethanol. This data was analysed separately to quantify the total number of nests and included only in the richness analyses.

For this study, only the ground-dwelling macroinvertebrate fauna (>2 mm) was included in the analysis to avoid potential biases when comparing data from burnt areas with unburnt areas due to the risk of smaller animals being totally consumed by the fire. All collected specimens were identified to Family level, except for the snails and ants that were identified to Order and Genus level, respectively. The latter was done because there are only a reduced number of ant sub-Families in Portugal (Harde and Severa 1984; Goulet and Huber 1993; Roberts 1995; Barrientos 1998; Czechowski et al. 2002). Snails were also an exception because of effects of the fire in their remains that in most cases only allowed Order level identification.

### Data Analysis

The diversity indices of abundance (*n*), taxon richness (*s*), Shannon-Weiner’s diversity index (H’), and Pielou’s equitability index (J’) were calculated for the individual sampled stones. The data distribution between burnt and unburnt areas was contrasted using boxplots. Living and dead animals collected in the burnt area were treated and plotted individually, being designated by Burnt_L and Burnt_D, respectively. No dead animals were found in the unburnt area. Differences in the abundance, richness, diversity and equitability between the (1) Unburnt, (2) Burnt L and (3) Burnt D were tested for statistical significance using the non-parametric Kruskal-Wallis test. Living and dead animal samples were analysed separately in the burnt area to verify mortality patterns and community differences that can be attributed to the wildfire between areas and within the burnt area.

In the case of significant differences, the contrasts between the individual treatments were tested for statistical significance using the post hoc Least Significant Difference (LSD) test. Non-parametric tests were preferred as the various data sets did not meet the ANOVA assumptions of normality and homoscedasticity, following the Shapiro-Wilk and Levene tests.

Sample-based species rarefaction (Mao’s tau) for the taxa with living specimens was assessed for the burnt and unburnt sites separately to estimate overall richness as a function of the number of samples (Colwell et al. 2004), allowing to attribute biodiversity losses to the wildfire. This analysis allowed to verify the suitability of the number of samples used in the experiment as sufficient to properly assess the ground-dwelling macroinvertebrate community related to stones. Dissimilarity estimation between potentially subsampled datasets (burnt area) was also allowed, providing insights into the potential richness of the overall population.

The variation in community composition among the 10 study sites was analyzed using non-metric multidimensional scaling (NMDS), combining the live animal data of all stones at each site. The measure of dissimilarity used in NMDS was Euclidean distance. In addition to NDMS, the differences in community composition between the burnt and unburn sites were tested for statistical significance using analysis of similarities (ANOSIM) with Euclidean distance as similarity index. NMDS was used to explore data among burnt and unburnt sites, while ANOSIM provided statistical evidence to support or reject hypotheses about differences in community composition between groups.

To evaluate the fire effects on community function, each taxon was classified according to their diet/feeding behaviour and habitat preferences. Based on the existing bibliography, the feeding habits were divided into five categories: predator, omnivore, herbivore, fungivore, and detritivore. Habitat associations were also divided into three categories based on the bibliography: ground-dwellers, underground-dwellers, and plant-dwellers. The distribution frequency among each class was then calculated using family richness and total abundance of live specimens in the unburnt and burnt areas. Chi-square tests were used to verify if the distribution of the feeding habits and habitat association of the ground-dwelling invertebrate community differed between the burnt and unburnt sites. If differences were found, a post hoc analysis was performed with adjusted residuals and a corrected Bonferroni p-value to identify which groups differed significantly between the classes of feeding habits and dominant habitat association. The Spearman rank correlation coefficient was used to quantify and test the relationship of the indices of abundance and richness with stone depth and area. The distribution of the ground cover classes in each unburnt and burnt site was obtained using the average class cover in each. All statistical tests used an alpha of 0.05 as an adequate and standardized significance level in these types of studies.

## Results

**Fig. 2:**
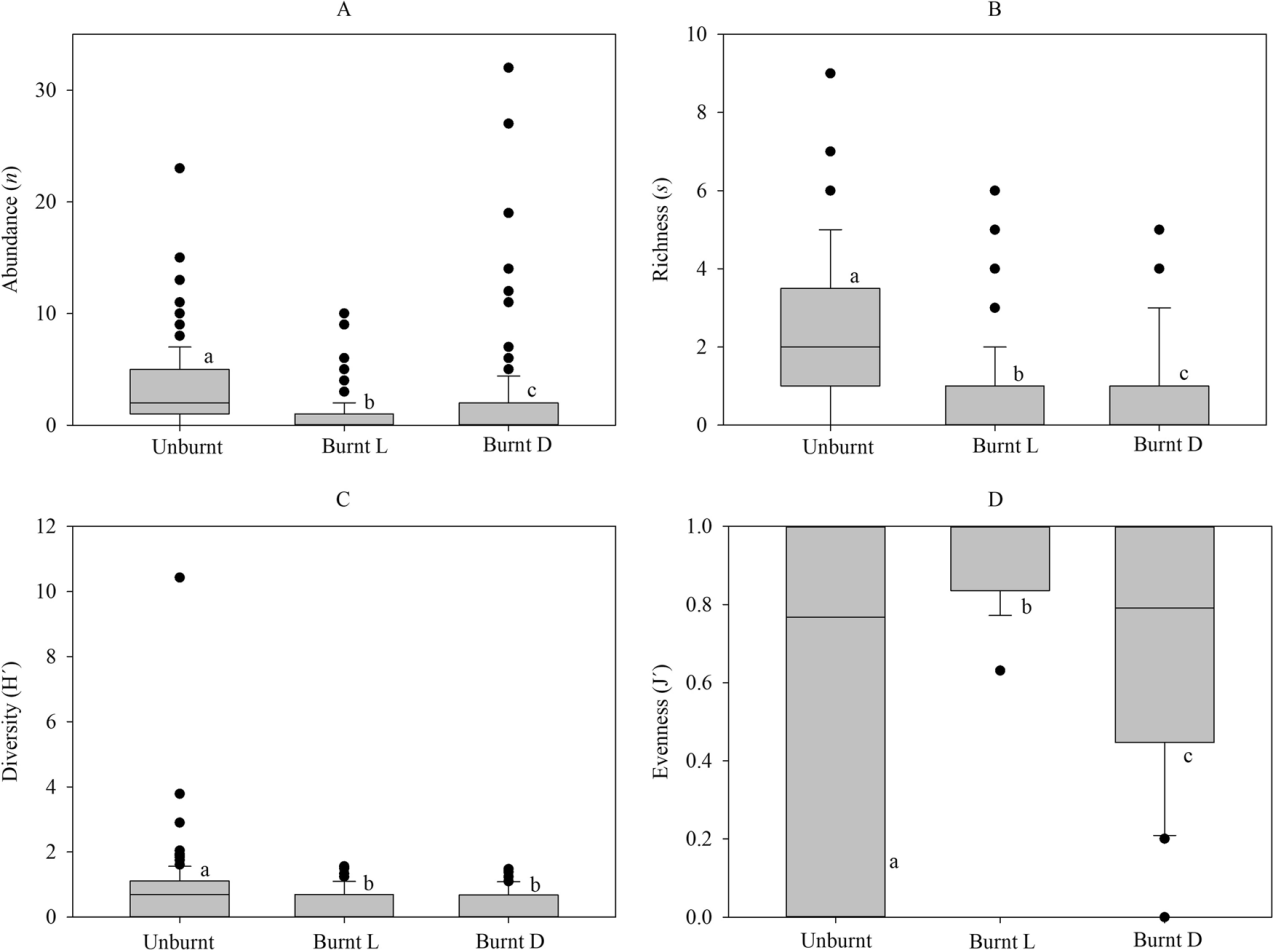
Abundance (n) (**A**), richness (s) (**B**), Diversity (H’) index (**C**) and Evenness (J’) index (**D**) of ground-dwelling invertebrates per stone (*n* = 165). Burnt L—live organisms collected in the Burnt area; Burnt D—dead organisms collected in the burnt area. Horizontal lines are the standard deviation. Distinct letters indicate significant differences between the three groups (*p* < 0.05)

The ground-dwelling macroinvertebrate community living under stones exhibited significant differences between burnt and unburnt areas across various parameters. These differences included abundance (H = 88.05, *p* < 0.001), richness (H = 100.9, *p* < 0.001), diversity (H = 54.28, *p* < 0.001), and evenness (H = 14.69, *p* < 0.001) (Fig. 2).

When comparing the abundance and richness values of alive invertebrates in both areas, significantly higher values were observed in the unburnt area (Abundance: H = 20.85, *p* < 0.001; Richness: H = 21.83, *p* < 0.001) (Fig. 2). Additionally, higher diversity values were found in the unburnt area (H = 19.15, *p* < 0.001), while significantly higher evenness values were noted in the burnt area (H = 11.97, *p* < 0.001) (Fig. 2).

A similar pattern to the one found in the unburnt area regarding alive animals was also observed in the burnt area, but for the dead animals collected. These had significantly higher values of abundance (H = 9.04, *p* < 0.01), richness (H = 17.03, *p* < 0.001), diversity (H = 21.77, *p* < 0.001), and evenness (H = 4.31, *p* < 0.05) (Fig. 2).

Regarding the abundance and richness of live (Burnt L) and dead (Burnt D) invertebrates in the burnt area on the same sample, slightly but significantly higher values were found in Burnt D for abundance (H = 5.89, *p* < 0.05) and richness (H = 5.69, *p* < 0.05) (Fig. 2). The diversity values were similar, with no significant differences between Burnt L and Burnt D sites (H = 3.93, *p* > 0.05). Evenness values were significantly higher in Burnt L (H = 3.91, *p* < 0.05) (Fig. 2).

**Fig. 3:**
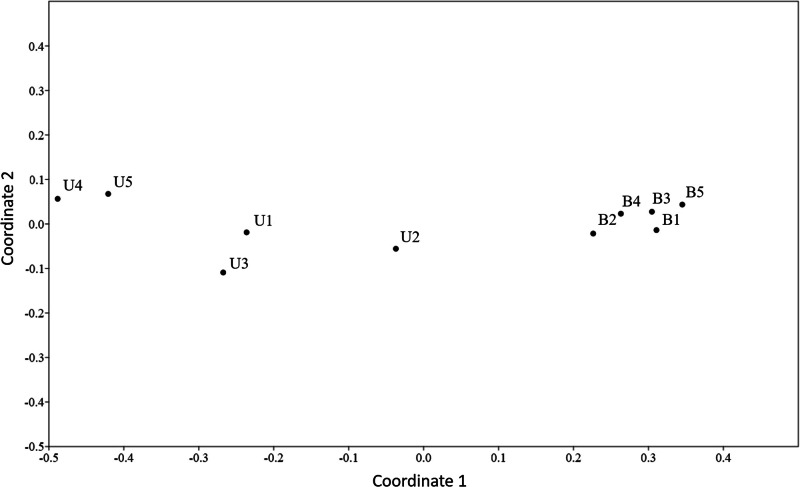
Non-metric multidimensional scaling (NMDS) plot for unburnt (U) and burnt (B) sites. Stress = 0.01

The NMDS showed segregation between unburnt and burnt sites (Fig. 3), and ANOSIM analysis confirms significant differences between the burnt and unburnt areas (R = 0.2366, *p* < 0.0001).

A total of 72 distinct invertebrate taxa were identified, with 40 exclusively found in the unburnt area and seven only in the burnt area. In the unburnt area, the total number of individuals found was 4038, while in the burnt area, it was 744. Ants living in nests constituted a significant portion of the organisms, with 3515 individuals in the unburnt area and 359 alive (251 ants in nests) and 385 dead (93 ants in nests) individuals in the burnt area.

**Fig. 4:**
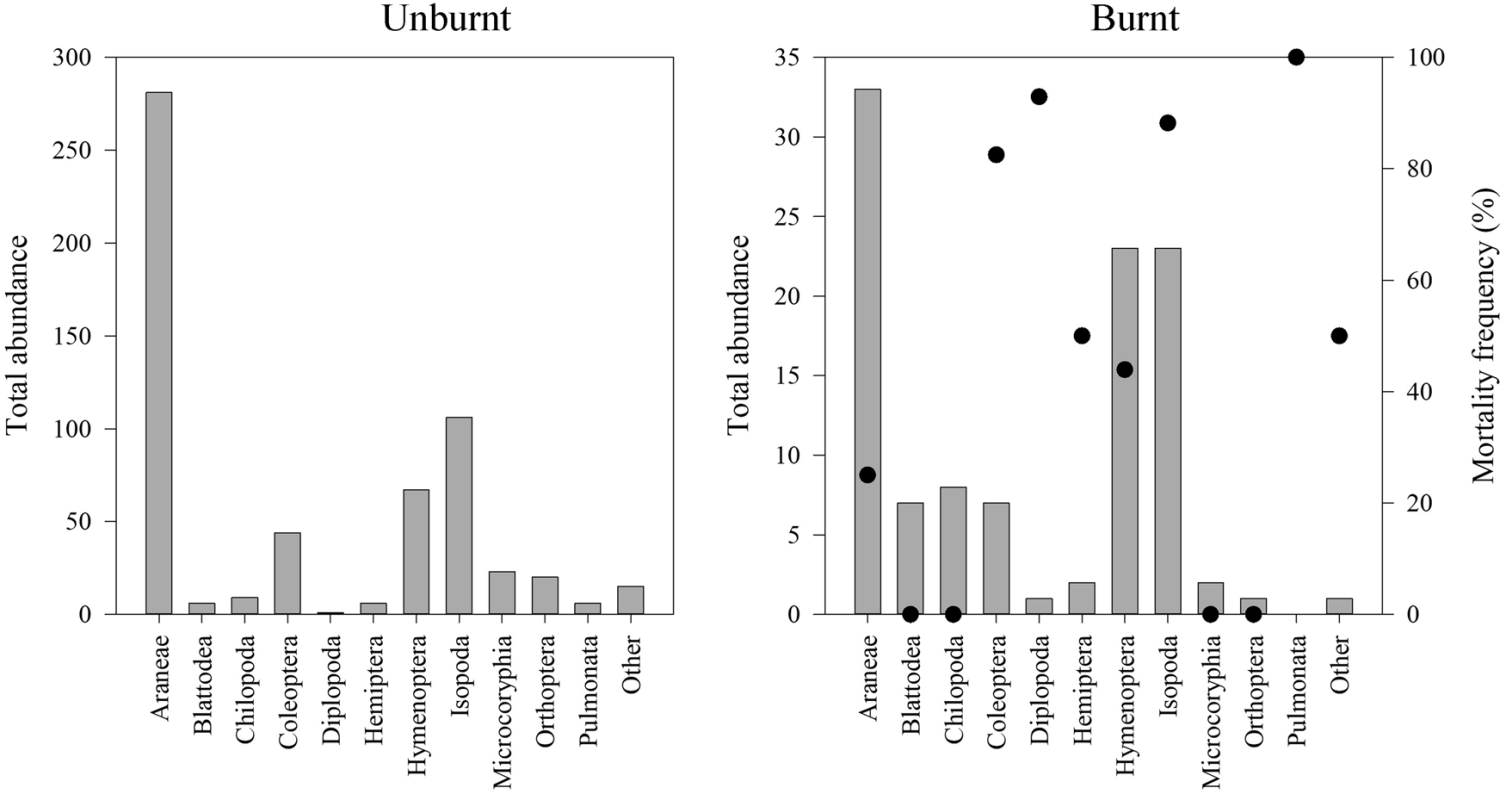
Total abundance per taxa (Order level), found in Unburnt and Burnt areas; Other taxa includes: Dermaptera; Lepidoptera, Neuroptera, Pseudoscorpionida, Thysanoptera, and Zygentoma; Black dots show the mortality frequency in each taxa

Alive specimens in both areas were dominated by Araneae, Hymenoptera, and Isopoda (Fig. 4). In the unburnt area, less numerous groups such as Coleoptera, Microcoryphia, and Orthoptera were observed, along with several less represented taxa (Appendix 1; Fig. 4). The burnt area exhibited dominance by the same groups as the unburnt area, with increased representativity of Blattodea and Chilopoda. No alive specimens were found in several groups in both areas, such as Lepidoptera, Zygentoma, Neuroptera, Pseudoscorpionida, Thysanoptera, and Pulmonata (Appendix 1).

**Fig. 5:**
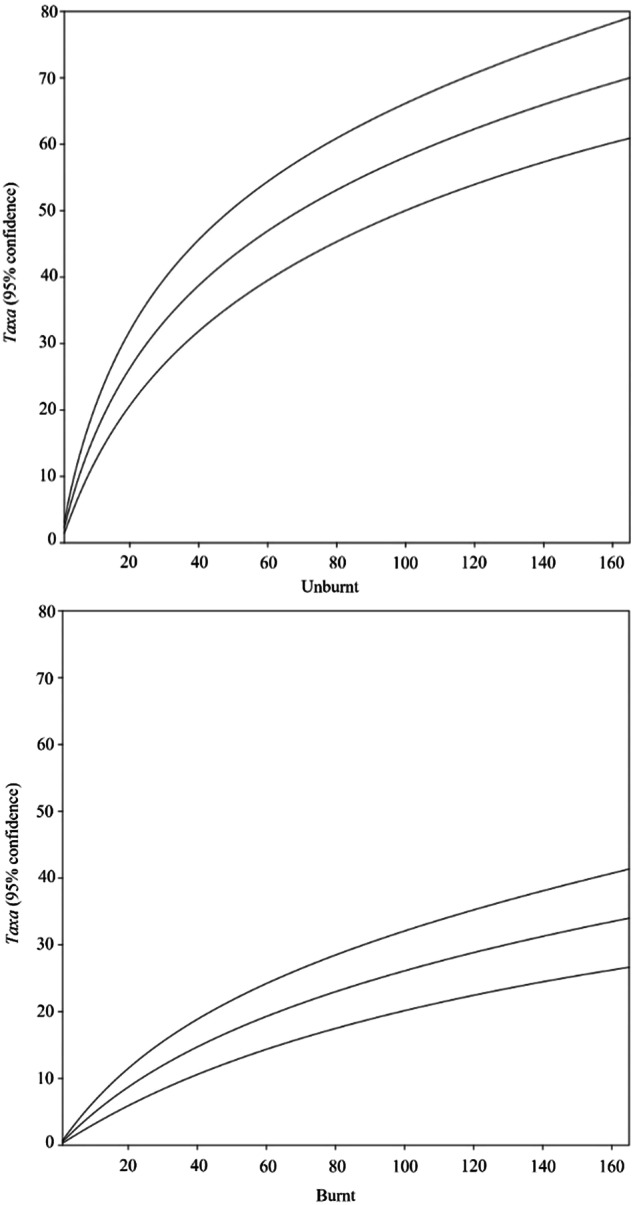
Sample-based rarefaction comparison between the unburnt and burnt areas (live taxa only). *n* = 165; upper and lower lines represent the 95% confidence intervals

Comparing the list of alive taxa (at the Order level) in the unburnt and burnt areas revealed that the most represented groups in the unburnt area still occurred in the burnt area, although their abundance was reduced (Fig. 4). The fire led to high mortality in all burnt sites, with more than half of the specimens exterminated. At the order level, Pulmonata, Isopoda, Diplopoda, and Coleoptera were the most affected taxa, with a mortality rate between 80% and 100%, while Araneae, Hemiptera, and Hymenoptera had less than 50% mortality. Microcoryphia was the least affected taxon. At the Family level (Appendix 1), a pronounced difference was observed between the areas, with 65 taxa found in the unburnt area and 29 in the burnt area. The number of families found in the burnt area was reduced, often to half or less of the number found under each stone in the unburnt area, as evident from the rarefaction analysis (Fig. 5). Despite a general decrease in biodiversity in most Orders in the burnt area compared to the unburnt area, seven taxa were identified only in the burnt area, corresponding to four Araneae, one Coleoptera, one Hemiptera, and one Isopoda family (Appendix 1).

**Fig. 6:**
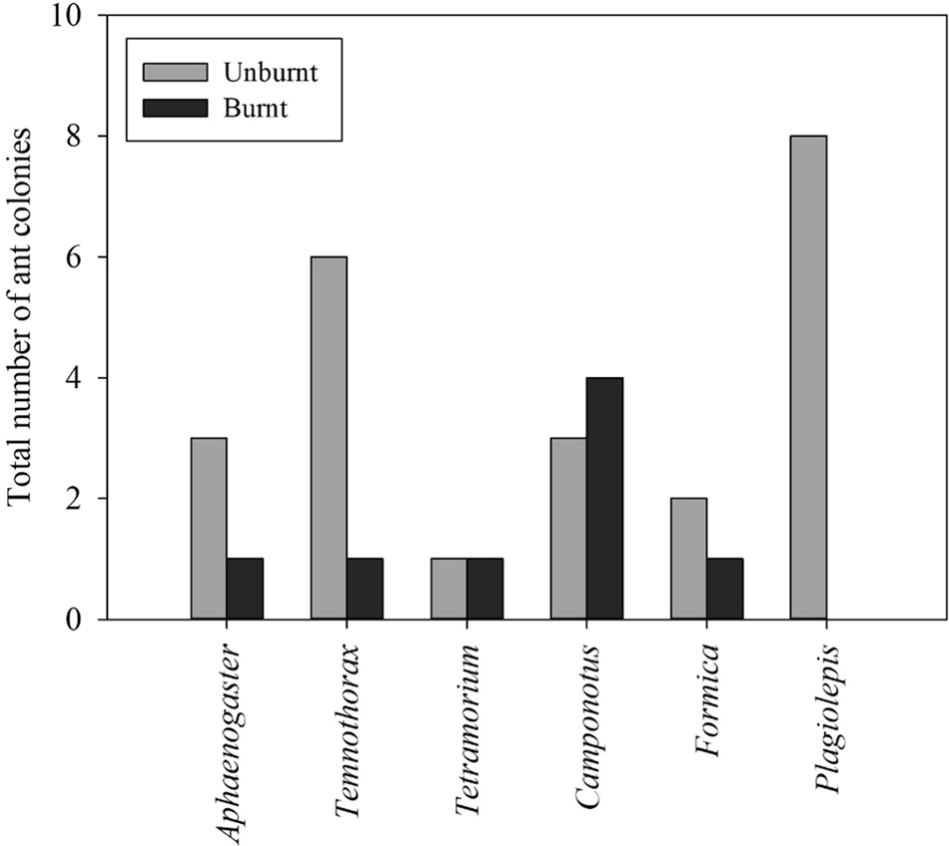
Total number of alive ant nests found (genus level) in unburnt and burnt areas

The number of alive ant nests found in the burnt area was lower than in the unburnt area for most identified taxa (Fig. 6), except for the Genus *Camponotus*. Genus *Plagiolepis*, despite being the most abundant in the unburnt area, was not found in the burnt area. ANOVA results showed significant differences in the number of ant nests found between areas (H = 3.692, *p* < 0.05).

### Immediate post-fire effects on community function

**Fig. 7:**
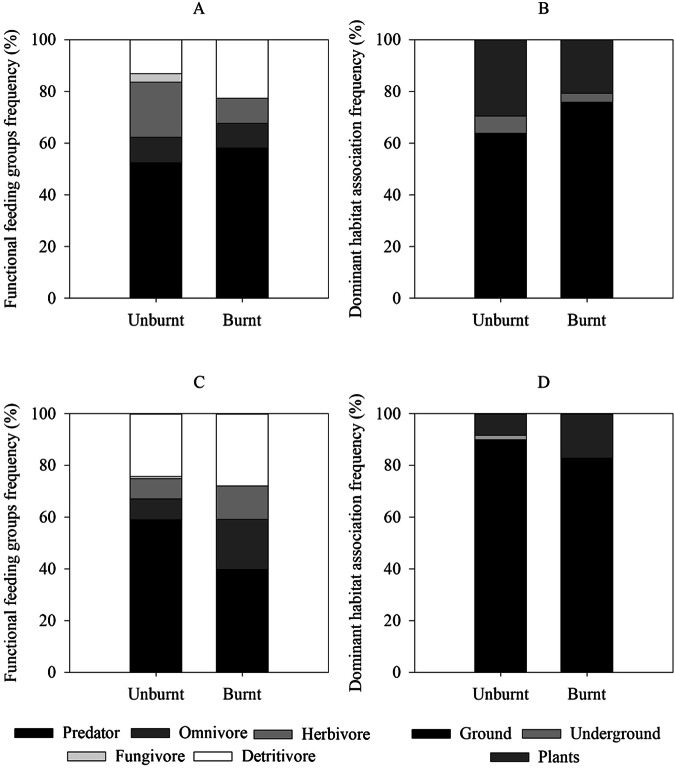
Ground-dwelling macroinvertebrate community functional feeding behaviour and dominant habitat distribution frequency at the family level richness (**A**, **B**) and for total abundance (**C**, **D**) in unburnt and burnt areas

Following the wildfire, immediate changes in functional feeding behaviour and habitat associations were noted in the burnt area, as depicted by family-level richness (Fig. 7A, B) and total abundance (Fig. 7C, D). Significant differences between areas were identified only in terms of diet (Chi-square = 18.963; *p* < 0.001) and habitat associations (Chi-square = 6.398; *p* < 0.05) concerning overall abundance.

In the burnt area, a decrease of approximately 20% in the number of predators and a complete absence of fungivores were observed. Conversely, a relative increase of 10%, 5%, and 3% was observed in the number of omnivores, herbivores, and detritivores, respectively. When analyzing at the family level, although no significant differences were observed, there was a 3% increase in predator taxa and an 11% increase in detritivore taxa. Meanwhile, there was an 11% decrease in herbivore taxa, and fungivores became non-existent, while the frequency of omnivore taxa remained unchanged.

Regarding the habitat association of the overall community, there was a 9% increase in plant-associated individuals observed in the burnt area compared to the unburnt area. Simultaneously, there was a 7% decrease in ground dwellers, and underground specimens became less than 1%. Family relative richness in the burnt area mirrored this trend, with ground dwellers dominating at 76%, followed by plant-associated taxa at 21%, and underground dwellers at 3%.

### Stone depth and stone area

**Fig. 8:**
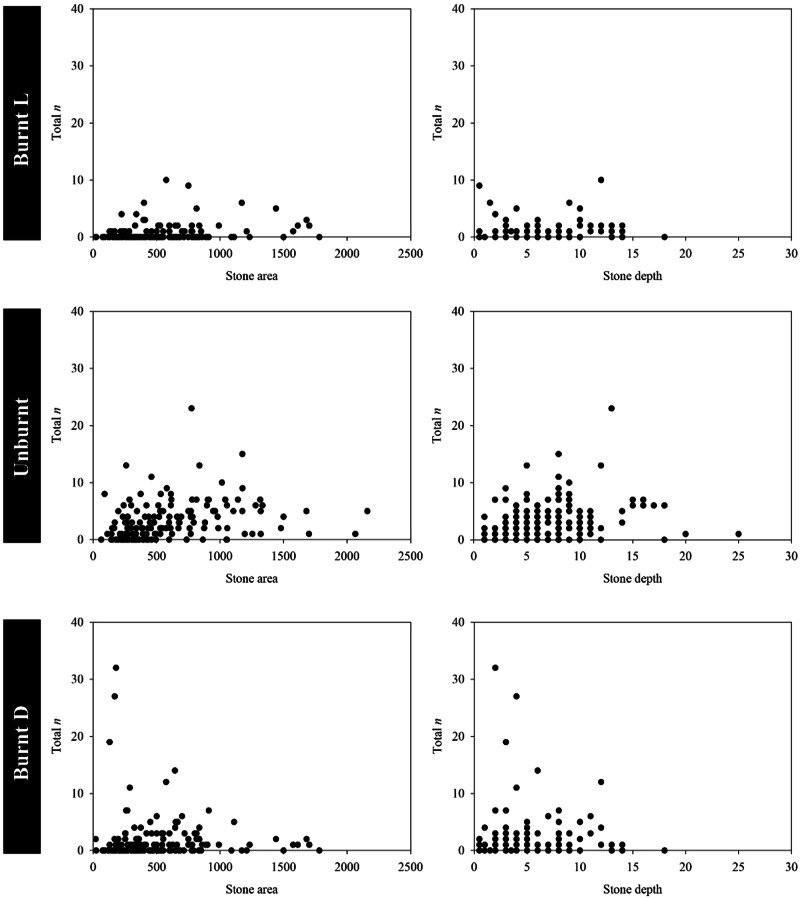
Total number of individuals (n) collected under each stone sampled according to stone area (left) and stone depth (right) in the unburnt and burnt areas. Burnt L—live organisms collected in the Burnt area; Burnt D—dead organisms collected in the burnt area

**Fig. 9:**
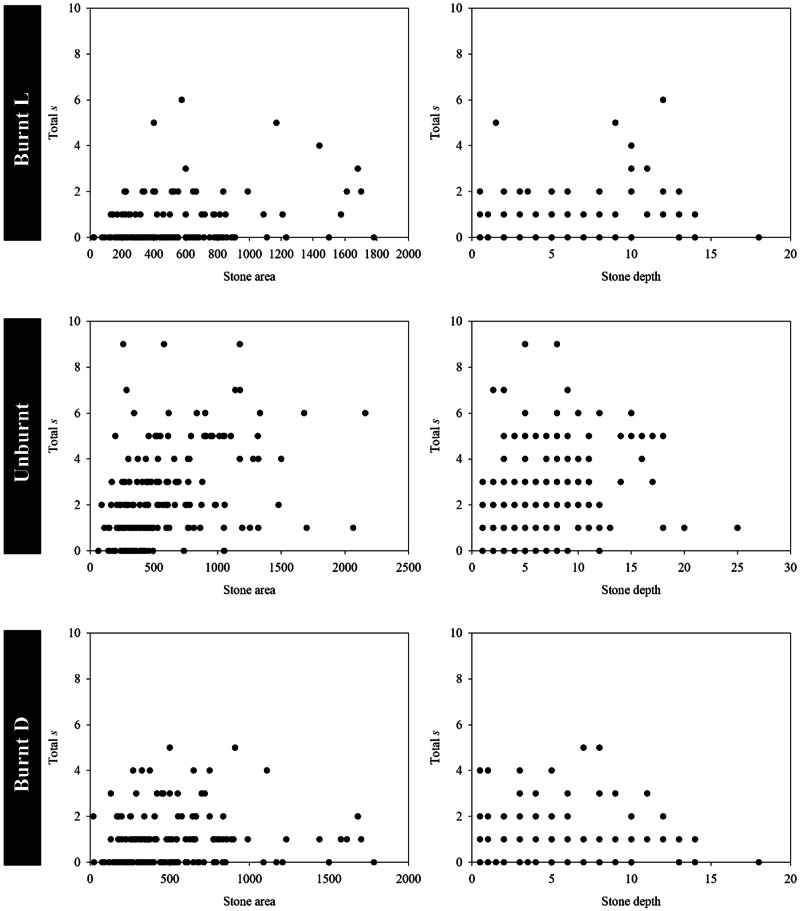
Total number of taxa (s) collected under each stone sampled according to stone area (left) and stone depth (right) in the unburnt and burnt areas. Burnt L—live organisms collected in the Burnt area; Burnt D—dead organisms collected in the burnt area

The features of the stones varied widely, ranging from 50 to 2160 cm^2^ in area and 0.5 to 25 cm in depth. Sampled stones, on average, had an area of 594 cm^2^ and a depth of 6.9 cm, whereas in the burnt area, the average area was 499 cm^2^, and the average depth was 5.1 cm (refer to Figs. 8, 9).

The occupancy rate of individuals beneath stones differed between unburnt and burnt areas. In the unburnt area, 85% of sampled stones harboured specimens, whereas in the burnt area, only 69% revealed individuals, with only 33% being alive. In the unburnt area, 92% of stones without individuals were those with an area less than 500 cm^2^, while in the burnt area, this percentage was 73%. Additionally, in the unburnt area, 15% of samples without arthropods were observed, of which 11% were buried above 5 cm in the ground. In the burnt area, this value increased to 49%. Significant differences were found in the unburnt area between stones with 0 individuals and those with at least one individual (Chi-square = 18.857; *p* < 0.001) for depths under 5 cm, regardless of stone area. In the burnt area, significant differences were found only between stones with 0 individuals and those with at least one individual (Chi-square = 15.436; *p* < 0.001) for stones with less than 500 cm^2^ and less than 5 cm depth, as well as for stones with more than 500 cm^2^ and more than 5 cm depth, specifically concerning live individuals.

In the associations between stone characteristics and ground-dwelling macroinvertebrates, it was found that deeper-buried stones were positively correlated with a high number of individuals (rs = 0.230; *p* < 0.05) and taxa (rs = 0.253; *p* < 0.05) in the burnt area. The same trend was observed for larger stones in the burnt area, which housed a high number of individuals (rs = 0.268; *p* < 0.05) and taxa (rs = 0.242; *p* < 0.05). Similarly, in the unburnt area, the relationship between stone depth and area, with the number of individuals and taxa, was also observed. A high number of individuals (rs = 0.231; *p* < 0.05) and taxa (rs = 0.183; *p* < 0.05) were exhibited by stones buried deeper in the soil. Likewise, a high number of individuals (rs = 0.169; *p* < 0.05) and taxa (rs = 0.168; *p* < 0.05) were also housed by larger stones.

## Discussion

Numerous factors can influence the direct impact of fire on the soil faunal community, including the fire’s characteristics, soil humidity, available vegetation, organic matter, and geography (Moreira et al. 2010; Pressler et al. 2018). However, microhabitats, particularly ground stones, have often been overlooked. This study sheds light on their pivotal role, revealing that stones serve as safeguard microhabitats and as refuges for the ground-dwelling macroinvertebrate community during catastrophic events like wildfires. Despite an overall decline in richness, abundance, and diversity due to fire-induced mortality, stones have proven instrumental in ensuring the survival of a wide range of taxa.

The community identified in burnt sites predominantly consisted of ground-dwelling groups, including Araneae, Hymenoptera (primarily ants), Coleoptera, and Isopoda. These findings align with other studies investigating post-fire effects on ground-dwelling invertebrates in the Mediterranean basin (Sgardelis et al. 1995; García-Domínguez et al. 2010; Puga et al. 2016) and other geographical regions (York 1999; Wikars and Schimmel 2001; Collet 2003; Coleman and Rieske 2006; Verble-Pearson and Yanoviak 2014; Kwok and Elridge 2015).

The wildfire had a significant impact on the diversity of ground-dwelling macroinvertebrates. Likewise, multivariate analysis effectively distinguished between unburnt and burnt sites. Soon after wildfire there is a decrease in the number of individuals and species in relation to unburned areas (Wikars and Schimmel 2001; Coleman and Rieske 2006; Verble-Pearson and Yanoviak 2014). Consequently, the direct consequences of the fire on the ground-dwelling macroinvertebrate community led to a substantial loss in diversity within the affected areas, reducing it under each stone to approximately half of the initial value encountered in non-affected areas. Sample-based rarefaction analysis corroborated this trend, emphasizing the disparity in diversity between the burnt and unburnt areas.

Despite the decrease in predator abundance and the decline of fungivores, followed by an uneven increase in the numbers of omnivores, herbivores, and detritivores, the functions of the community were not compromised by the wildfire. This resilience was evident as most Orders identified in the unburnt area continued to be represented in the burnt area, even though there was an overall reduction in the number of families and diminished equitability. Supporting these findings, Kaynas (2016) reported identical abundance and richness results in surface-dwelling arthropod communities of Anatolian pine forests a month after a fire. Mason et al. (2021) further substantiated these outcomes, with several examples of invertebrate resilience post-fire and adaptation to changes in the ecosystem over time.

In terms of mortality, our findings suggest that wildfire events can directly impact most taxa constituting the community beneath stones in native oak forests, with a mortality rate exceeding 50% in the burned sites within this specific community. Similar observations were made in Europe by Wikars and Schimmel (2001), who noted a high mortality among soil invertebrates in boreal pine forests affected by wildfires. This pattern was also evident in different Italian native forest types of *Quercus*, *Pinus*, and *Populus*, as observed by Trucchi et al. (2009), and in Greek native phryganic vegetation ecosystems, as reported by Sgardelis et al. (1995).

Mortality displayed variations across the community, with some groups experiencing high rates, while for several other taxa, the direct effects of the fire were less pronounced. As observed in other studies, animals with traits that make them highly vulnerable to fire, such as slow locomotion, moisture dependency, and larger body size, tended to have higher mortality rates, as previously noted by Moretti and Legg (2009) and Trucchi et al. (2009). Accordingly, the ground-dwelling community can be categorized into three groups based on their resilience to fire.

The first group, with lower mortality rates when compared to the total abundance values found in the unburnt area, includes cockroaches (Blattodea), centipedes (Chilopoda), rock bristletails (Microcoryphia), and crickets (Orthoptera). Abbott et al. (2003) reported rapid recovery of cockroach and cricket populations from fire events, results also obtained by Arnold et al. (2016), who suggested that cockroaches can survive wildfires despite a reduction in diversity compared to areas outside the burnt area. Our study supports the results of Trucchi et al. (2009), who found that despite changes in composition, centipedes could survive fires in deeper soil layers. Rock bristletails, although information is limited, appear to be the least affected among all groups when comparing total abundance values between unburnt and burnt areas. As suggested by Lewis (1981) for centipedes, our field observations at the time of collection indicate that rock bristletails can benefit from aestivation behaviour by seeking shelter under deeper-buried stones.

The second group comprises spiders (Araneae) and ants (Hymenoptera), with a high mortality rate; however, the abundance of live animals in the burnt area remained high compared to other taxa. Nevertheless, the richness and abundance of spiders decreased in the burnt area, in line with observations by Wikars and Schimmel (2001) and Verble-Pearson and Yanoviak (2014). In our study, spider families associated with vegetation were most affected, with several taxa absent from the burnt area. Most remaining spiders in the burnt area were ground-dwellers, using stones as hunting and nesting grounds. Behavioural considerations may have been a decisive factor in the differences in mortality among Araneae. Similar results for spiders in rocky fynbos habitats three months after a fire were found by Yekwayo et al. (2019), where a decrease in abundance and richness was observed, and survivability was attributed to potential refuges such as rocks and plants, as suggested by Pryke and Samways (2012).Regarding ants, both wandering individuals and ant nests suffered a significant reduction in their numbers across all inventoried genera. *Plagiolepis* sp. nests were absent from the burnt area, despite being the most abundant in the unburnt area. This outcome raises questions about the wildfire’s ability to alter the ground-dwelling macroinvertebrate community, whether temporarily or more permanently, particularly in areas subject to repeated wildfires, warranting special attention regarding conservation and management issues. Previous studies focusing on fire effects on ants have demonstrated that such community shifts occur, favouring open land ant species at the expense of forest specialist ants (Andersen et al. 2009; Matsuda et al. 2011; Anjos et al. 2017). The absence of wandering individuals of *Crematogaster* sp. in the burnt area supports this assumption, as this taxon is associated with vegetation and commonly builds nests in trees.

A third group, characterized by the highest rates of mortality, includes the Orders Pulmonata, Isopoda, and Diplopoda, all of which exhibit high moisture dependence, with the first having slow locomotion, rendering them more susceptible to the direct effects of fire. Similar findings were reported by Moretti et al. (2004), identifying a reduction in isopod richness and abundance in burnt areas, and by Sgardelis et al. (1995), who observed a decrease in Isopoda and Diplopoda abundance post-fire, with the latter persisting for at least two years since the fire. The immediate post-fire effects on snail populations, as noted by Kiss and Magnin (2006), can drastically reduce their abundance and richness. However, land snail populations seem resilient to fire events, partly due to the presence of refuges facilitating their initial survival (Nekola 2002; Kiss et al. 2004; Kiss and Magnin 2006). Despite the ecological value of stones as microhabitats, the notably high number of isopods recorded (both dead and alive) in the burnt area compared to the unburnt area suggests that these animals, aside from using stones for nesting and foraging, might have sought refuge under stones during the wildfire. This behaviour could explain the unusually high number of dead isopods found under some of the sampled stones. This search for protection under stones during fire events may have also influenced the mortality of Coleoptera, seemingly associated with body size, as mortality primarily occurred in larger specimens closer to the outer perimeter of the sampled stones. Prior studies on immediate post-fire effects on ground-dwelling coleopterans have demonstrated negative impacts on both abundance and richness (Wikars and Schimmel 2001; Moretti et al. 2004), followed by a shift in composition favouring pyrophilous species (Sasal et al. 2010; Fredriksson et al. 2020).

Furthermore, considering the relationship between mortality and body size, it is crucial to acknowledge that smaller individuals, regardless of taxon, may have been entirely consumed by the fire. Consequently, the impacts of the wildfire on the lethality of ground-dwelling invertebrates associated with stones may have been underestimated and this potential issue should be addressed in future studies.

Regarding the characteristics of stones, the abundance and richness of ground-dwelling organisms exhibit an upward trend with larger stones and greater depths in both areas. Larger stones, in terms of habitat, foster a higher number of individuals and species, thereby contributing to increased biodiversity. Greater depths provide an opportunity for the coexistence of additional ecological niches and strategies that promote a rise in species numbers. Furthermore, the larger and more deeply buried a stone is in the soil, the more effectively it shields against fire. Notably, depths ranging from 5 to 15 cm appear to present a greater likelihood of encountering live specimens compared to depths beyond this range. These findings align with existing studies indicating that the direct effects of fire can be negligible a few centimetres below the soil surface, as the elevated temperatures above the soil tend to dissipate and rapidly decrease (New 2014; DeBano 2000; Moreira et al. 2010; Caut et al. 2013). In terms of stone area, it is reasonable to assume that the larger the stone, the more isolated the centre of each stone will be from the fire, providing a safer area for animals living or seeking refuge underneath.

As noted in prior research, refuges play a crucial role for many species during catastrophic events (Brennan et al. 2011; Gongalsky et al. 2012; Pryke and Samways, 2012). Examining the ground-dwelling invertebrate community associated with stones in Mediterranean oak forests, this study unveils a diverse and extensive community, considering the average size of stones in the forest ground. These microhabitats support numerous invertebrate species, primarily ground dwellers, incorporating stones into their ecological requirements. Our findings present evidence of stones serving as nesting sites for various spider families (e.g., Oecobiidae, Dysderidae, Zoridae), woodlice (Stenoniscidae, Cylisticidae, and Porcellionidae), rock bristletails (Meinertellidae), silverfish (Nicoletiidae), and for most identified ant and termite taxa.

Moreover, our data reveals that stones are utilized as hunting grounds for many predators, predominantly spiders and centipedes (Lithobiidae and Geophilidae), and as foraging grounds for cockroaches (Ectobiidae), woodlice, rock bristletails, silverfish, crickets (Gryllidae), and ground beetles (Staphylinidae, Carabidae, Scarabidae, and Tenebridae). Stones also appear to function as aestivation sites for Geophilidae and Meinertellidae species. These taxa collectively form the core community associated with stones in oak forests, prevailing even after a wildfire. Predators, particularly ground spiders, both in terms of abundance and diversity, dominate the invertebrate community directly linked with stones emphasizing their importance in this microhabitat.

Additionally, various taxa find temporary refuge under stones, although their association is commonly linked to plants and vegetation. These taxa, including true bugs, moths, and beetles, exhibit lower abundances compared to the previously described groups.

The wildfire-induced destruction of vegetation and litter diminishes the number of plant and litter-associated taxa, as they are also consumed by the fire. Only a few individuals belonging to families typically associated with plants, such as Oxyopidae, Philodromidae spiders, Cucujidae beetles, or Lygaeidae bugs, were found under stones, suggesting that these animals actively sought refuge beneath the stones as a survival strategy.

Despite the mortality and the reduced abundance and diversity, many taxa persisted in the burnt area. This suggests that post-fire recolonization, coupled with the influx of organisms from peripheral unburnt areas, as proposed in previous studies (Zaitsev et al. 2014; Yekwayo et al. 2016; Swart et al. 2017), may also stem from refuge sites within the burnt area, such as stones.

This study emphasizes the pivotal role of stones in promoting the survival of ground-dwelling organisms during wildfires, highlighting their particular significance as refuges. This information holds importance, as a lack of adequate evidence on invertebrate diversity and ecology, especially their responses to fire, hampers the ability to make evidence-informed decisions regarding post-fire land restoration and research funding (Saunders et al. 2021).

## Conclusions

The main conclusions of the present study were the following:

1. Ground stones, often overlooked, play a pivotal role as safeguard microhabitats and refuges for the ground-dwelling macroinvertebrate community during wildfires.
2. The community associated with stones is predominantly composed of ground-dwelling groups (Araneae, Hymenoptera, Coleoptera, Isopoda) before and after the wildfire, aligning with consistent patterns observed in post-fire effects across different geographical regions.
3. Wildfires significantly impact the diversity of ground-dwelling macroinvertebrates, leading to an overall decline in richness, abundance, and diversity shortly after the event.
4. Despite declines in predator abundance, the community’s functions remain uncompromised, showing resilience. Most orders from unburnt areas are still represented in burnt areas, highlighting adaptability to post-fire ecosystem changes.
5. Wildfires directly impact taxa under stones, resulting in a mortality rate exceeding 50%, with varied patterns across groups based on vulnerability traits.
6. Stone size and depth play a crucial role in promoting survival during wildfires.
7. Stones serve as essential refuges for a diverse invertebrate community during and after wildfires. Evidence showed that several taxa actively took refuge under stones, contributing to post-fire recolonization despite the overall impacts on abundance and diversity.
8. This study emphasizes the importance of stones in promoting ground-dwelling organism survival during wildfires. This information is crucial for informed decisions on post-fire land restoration and research funding, addressing the information gap on invertebrate diversity and ecology responses to fire.

## Supplementary Information

Appendix

## Data Availability

The data described in this article will be available on www.figshare.com upon article publication.
