# Supplementary material for: Immediate Impacts of Wildfires on Ground-dwelling macroinvertebrate Communities under Stones in Mediterranean Oak Forests
Source: Environ Manage. 2024 Jun 21;74(4):684–98. doi: 10.1007/s00267-024-02006-z (PMC11392986; doi:10.1007/s00267-024-02006-z)
Supplement: Supplementary file 1 — Appendix [file 267_2024_2006_MOESM1_ESM.docx]

**Table 1** Occurrence, dominant feeding behavior and habitat associations of each *taxa* in U (Unburnt) and B (Burnt) areas.

| **Order** | **Family** | **U** | **B*** | **Dominant feeding behavior** | **Dominant habitat association** | | |  |
| --- | --- | --- | --- | --- | --- | --- | --- | --- |
|  |  |  |  |  | **Ground** | **Underground** | **Plants** |  |
|  |  |  |  |  |  |  |  |  |
| **Araneae** | **Agelenidae** | • | • | Predator | • |  | • |  |
|  | **Amaurobiidae** | • |  | Predator | • |  |  |  |
|  | **Anyphaenidae** | • |  | Predator |  |  | • |  |
|  | **Araneidae** | • |  | Predator |  |  | • |  |
|  | **Corinnidae** | • |  | Predator | • |  |  |  |
|  | **Dictynidae** | • |  | Predator | • |  | • |  |
|  | **Dysderidae** | • | • | Predator | • |  |  |  |
|  | **Gnaphosidae** | • | • | Predator | • |  |  |  |
|  | **Hahniidae** | • | • | Predator | • |  | • |  |
|  | **Linyphiidae** | • |  | Predator | • |  |  |  |
|  | **Liocranidae** | • |  | Predator | • |  |  |  |
|  | **Lycosidae** | • |  | Predator | • |  |  |  |
|  | **Mimetidae** | • |  | Predator |  |  | • |  |
|  | **Oecobiidae** | • |  | Predator | • |  |  |  |
|  | **Oonopidae** | • |  | Predator | • |  |  |  |
|  | **Oxyopidae** |  | • | Predator |  |  | • |  |
|  | **Philodromidae** |  | • | Predator |  |  | • |  |
|  | **Pisauridae** | • | • | Predator | • |  | • |  |
|  | **Salticidae** | • |  | Predator | • |  | • |  |
|  | **Scytodidae** |  | • | Predator | • |  |  |  |
|  | **Segestriidae** | • |  | Predator | • |  |  |  |
|  | **Sparassidae** | • |  | Predator |  |  | • |  |
|  | **Theridiidae** | • |  | Predator | • |  |  |  |
|  | **Thomiisidae** | • |  | Predator |  |  | • |  |
|  | **Zodariidae** |  | • | Predator | • |  |  |  |
|  | **Zoridae** | • | • | Predator | • |  |  |  |
| **Blattodea** | **Ectobiidae** | • | • | Omnivore | • |  | • |  |
|  | **Rhinotermitidae** | • |  | Detritivore | • | • |  |  |
| **Chilopoda** | **Lithobiidae** | • | • | Predator | • |  |  |  |
|  | **Geophilidae** | • |  | Predator |  | • |  |  |
|  | **Scutigeridae** | • |  | Predator | • |  |  |  |
| **Coleoptera** | **Anthribidae** | • |  | Fungivore | • |  |  |  |
|  | **Carabidae** | • | • | Predator | • |  | • |  |
|  | **Chrysomelidae** | • | • | Herbivore |  |  | • |  |
|  | **Cucujidae** |  | • | Predator |  |  | • |  |
|  | **Curculionidae** | • |  | Herbivore |  |  | • |  |
|  | **Elateridae** | • |  | Herbivore | • | • |  |  |
|  | **Endomychidae** | • |  | Fungivore | • |  |  |  |
|  | **Meloidae** | • |  | Herbivore |  |  | • |  |
|  | **Scarabidae** | • |  | Detritivore | • | • |  |  |
|  | **Staphylinidae** | • |  | Predator | • |  |  |  |
|  | **Tenebridae** | • | • | Omnivore | • |  |  |  |
| **Diplopoda** | **Julidae** | • | • | Detritivore | • | • |  |  |
| **Hemiptera** | **Acanthosomidae** | • |  | Herbivore |  |  | • |  |
|  | **Anthocoridae** | • |  | Predator |  |  | • |  |
|  | **Aphidoidea** | • |  | Herbivore |  |  | • |  |
|  | **Lygaeidae** |  | • | Herbivore |  |  | • |  |
|  | **Miridae** | • |  | Herbivore |  |  | • |  |
|  | **Nabidae** | • |  | Predator |  |  | • |  |
| **Hymenoptera** | ***Aphaenogaster*** | • | • | Predator | • | • |  |  |
|  | ***Crematogaster*** | • |  | Predator | • |  | • |  |
|  | ***Messor*** | • |  | Herbivore | • |  |  |  |
|  | ***Temnothorax*** | • | • | Herbivore | • |  | • |  |
|  | ***Tetramorium*** | • | • | Predator | • |  |  |  |
|  | ***Camponotus*** | • | • | Omnivore | • |  |  |  |
|  | ***Formica*** | • | • | Predator | • |  |  |  |
|  | ***Lasius*** | • |  | Omnivore | • |  |  |  |
|  | ***Plagiolepis*** | • |  | Omnivore | • |  |  |  |
|  | **Ichneumonidae** | • |  | Herbivore |  |  | • |  |
| **Isopoda** | **Cylisticidae** | • | • | Detritivore | • | • |  |  |
|  | **Porcellionidae** | • | • | Detritivore | • | • |  |  |
|  | **Stenoniscidae** | • | • | Detritivore | • | • |  |  |
|  | **Trichoniscidae** |  | • | Detritivore | • | • |  |  |
| **Lepidoptera** | **Gelechiidae** | • |  | Herbivore |  |  | • |  |
| **Microcoryphia** | **Meinertellidae** | • | • | Detritivore | • | • |  |  |
| **Zygentoma** | **Nicoletiidae** | • |  | Detritivore | • | • |  |  |
| **Neuroptera** | **Myrmeleontidae** | • |  | Predator | • |  |  |  |
| **Orthoptera** | **Gryllidae** | • | • | Omnivore | • | • |  |  |
| **Pseudoscorpionida** | **Chthoniidae** | • |  | Predator | • | • |  |  |
|  | **Garypidea** | • |  | Predator | • | • |  |  |
| **Thysanoptera** | **Thripidae** | • |  | Herbivore |  |  | • |  |
| **Pulmonata** | | • |  | Herbivore | • |  | • |  |

* Only alive specimens; Harde & Severa, 1984; Barrientos, 1988; Goulet et Huber, 1993; Buddle *et al*., 2000; Czechowski *et al*., 2002; Collet, 2003: Moretti *et al*., 2006; Andersen *et al*., 2009; Gongalsky *et al*., 2012; Kim & Holt, 2012; New, 2014; Lissner, 2014; Zaitsev *et al*., 2014; Barton *et al*., 2017; Nentwig *et al*., 2021; Oger, 2021
